# Hippocampal neural stem cell–derived extracellular vesicles modulate microglia to promote resilience against tau oligomers

**DOI:** 10.4103/NRR.NRR-D-25-00195

**Published:** 2025-11-25

**Authors:** Salvatore Saieva, Pietro Scaduto, Anna Fracassi, Jutatip Guptarak, Wen-Ru Zhang, Kathia Johnson, Daniel C. Jupiter, Michela Marcatti, Olga Zolochevska, Giulio Taglialatela, Maria-Adelaide Micci

**Affiliations:** 1 Mitchell Center for Neurodegenerative Diseases, Department of Neurology, The University of Texas Medical Branch, Galveston, TX, USA; 2 Department of Anesthesiology, The University of Texas Medical Branch, Galveston, TX, USA; 3 Department of Biostatistics & Data Science, Department of Orthopaedic Surgery and Rehabilitation, The University of Texas Medical Branch (UTMB), Galveston, TX, USA

**Keywords:** Alzheimer’s disease, hippocampus, immune response, lysosomes, microglia, neural stem cells, neuroinflammation, single-nuclei RNA sequencing, small extracellular vesicles, Tau oligomers

## Abstract

Neural stem cells and adult hippocampal neurogenesis modulate synaptic plasticity and cognitive function. Neural stem cells secrete extracellular vesicles – microvesicles carrying biomolecular cargos – that modulate the function of other cells and contribute to homeostasis and plasticity in the central nervous system. Alzheimer’s disease is marked by a reduction of neural stem cells in the hippocampus dentate gyrus. While increased neural stem cells often correlate with better learning and memory, neurogenesis alone does not always preserve these processes, indicating that other mechanisms involving neural stem cells support memory. It has been shown that intracerebroventricular delivery of neural stem cell-derived small extracellular vesicles in wild-type mice reduces cognitive decline and toxic oligomer binding to synapses. We hypothesize that adequate neural stem cell numbers support neural stem cell–derived small extracellular vesicles protection of synapses against Alzheimer’s disease toxic oligomers. Here, we show that elements of immune response in the central nervous system, particularly microglia, may contribute to this protective effect. Specifically, fluorescence-labeled small extracellular vesicles injected into wild-type mice brains were taken up by microglia, with only neural stem cell–derived small extracellular vesicles causing increased microglial activation, indicated by CD68 immunostaining. RNA-sequencing data showed selective activation of immune pathways in microglia by neural stem cell-derived small extracellular vesicles, leading to greater activation and higher Tau uptake 24 hours post-neural stem cell–derived small extracellular vesicle administration. Single-nuclei RNA-sequencing of hippocampal microglia gene revealed modulation related to lysosomal activity, supporting neural stem cell–derived small extracellular vesicle-induced neuroprotection via microglia. This study uncovers a novel mechanism through which neural stem cell–derived small extracellular vesicles enhance microglial activity and provide neuroprotection in the hippocampus. Our data demonstrates that neural stem cell–derived small extracellular vesicle uptake by microglia leads to increased microglial activation and improved uptake of Tau oligomers by microglia, suggesting that neural stem cell–derived small extracellular vesicles may prime microglia for a more effective immune response. These results support the hypothesis that neural stem cell–derived small extracellular vesicle-induced modulation of microglial function is crucial for preserving neuronal integrity and mitigating neurodegenerative processes. By elucidating the interactions between neural stem cell–derived small extracellular vesicles and microglia, our study opens new avenues for developing therapeutic strategies aimed at boosting microglial function and addressing neurodegenerative diseases such as Alzheimer’s disease.

## Graphical abstract

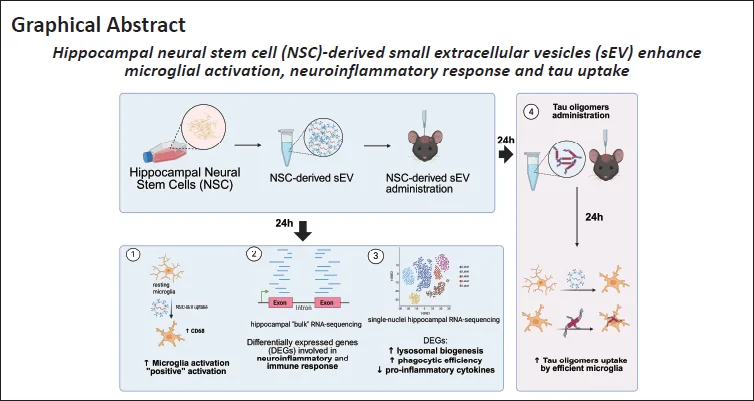

## Introduction

Alzheimer’s disease (AD) is the most common neurodegenerative disease, characterized by progressive loss of memory and cognitive decline (Colom-Cadena et al., 2023; Wang et al., 2024). However, no effective and safe cure exists yet, thus prompting researchers to explore alternative therapeutic approaches (Reuben et al., 2024).

A promising approach stems from the non-demented individuals with Alzheimer’s neuropathology (NDAN), who show intact memory performance, while displaying AD-like brain pathology (Bjorklund et al., 2012). NDANs show little synapse loss (Bjorklund et al., 2012), unique proteome signature compared with AD patients (Zolochevska et al., 2018), resistance to amyloid-beta (Aβ) and Tau oligomers (TauO) binding to synapses (Bjorklund et al., 2012; Singh et al., 2020), more efficient phagocytic microglia (Fracassi et al., 2023), preserved autophagy elements (Tumurbaatar et al., 2023), and preserved neurogenesis (Briley et al., 2016).

Neurogenesis, the process leading to the formation of new neurons from neural stem cells (NSC), occurs in the adult mammalian brain in the subventricular zone (SVZ) and the hippocampus dentate gyrus (DG). While neurogenesis typically declines with age (Moreno-Jiménez et al., 2021; Zhou et al., 2022) it declines more rapidly in AD patients (Choi and Tanzi, 2023; Cao et al., 2024). Notably, while compelling evidence in animal models (Van Praag et al., 2005; Connolly et al., 2022; Pan et al., 2025) and NDANs (Briley et al., 2016) highlights that a high number of NSC in the DG correlates with preserved memory and cognitive function, the number of mature neurons (MN) does not correlate with preserved cognition (Briley et al., 2016). Therefore, the mechanism linking the number of NSC in the hippocampus with preserved memory function remains elusive.

NSC secrete large amounts of small extracellular vesicles (sEV), which mediate many actions attributed to NSC (Bian et al., 2020; Hou et al., 2020) and are promising potential candidates for neurodegenerative therapies (Vogel et al., 2018). sEV are a subset of extracellular vesicles ranging between 40 and 160 nm in size (Chen et al., 2024), released by all cells. They carry biomolecules whose composition differs in a cell-specific manner (Mendonca et al., 2025). One of the major biomolecules carried by sEV is microRNA (miRNA), small non-coding single-stranded RNA filaments that silence gene expression at post-transcriptional level by degrading or translationally repressing multiple mRNA (Ma and Zhao, 2023; de Souza et al., 2025). Previous studies show that neural stem cell-derived-small extracellular vesicles (NSC-sEV), via their microRNA cargo, prevent Aβ oligomers binding to synapses, and associated synaptic and cognitive dysfunction, suggesting a key role of NSC-sEV in inducing synaptic resilience against AD pathology (Briley et al., 2016; Micci et al., 2019; Zolochevska and Taglialatela, 2020; Krishnan et al., 2025). Moreover, it has been reported that sEV released by NSC in the SVZ can regulate microglia morphology and modulate pathways associated with inflammatory response, cytokine regulation, and chemotaxis (Morton et al., 2018). Interestingly, NDANs exhibit a more efficient microglial activation, leading to a more competent phagocytic activity, thus suggesting that NDANs may have a unique type of microglia that eliminates impaired synapses, ultimately conferring resilience and maintaining cognitive integrity (Fracassi et al., 2023).

Here, we aimed to determine whether hippocampal NSC-sEV, compared with MN-derived small extracellular vesicles (MN-sEV), modulate microglial activity and immune responses. Specifically, we investigated whether NSC-sEV are preferentially taken up by microglia, induce their activation, and enhance their capacity to clear toxic TauO. Using complementary approaches — including fluorescent sEV tracking, bulk and single-nuclei RNA sequencing — we identify distinctive NSC-sEV effects on microglial lysosomal and neuroinflammatory pathways, highlighting a novel mechanism through which NSC-sEV promote hippocampal neuroprotection and synaptic resilience.

## Methods

### Animals

Mice (C57BL/6 J; 6–8 weeks old, 20–25 g of body weight, strain number 000664, RRID: IMSR_JAX:000664) were purchased from Jackson Laboratories (Bar Harbor, ME, USA, https://www.jax.org/strain/000664). A total of 54 mice were used across different experiments: 17 for sEV tracking (*n* = 3 naïve, *n* = 4 phosphate-buffered saline (PBS; Corning, Manassas, VA, USA, Cat# 21-031-CV), *n* = 5 NSC-sEV, *n* = 5 MN-sEV), 7 for single nuclei RNA-sequencing (snRNA-seq) (*n* = 4 NSC-sEV, *n* = 3 MN-sEV), 18 for RNA-bulk sequencing (*n* = 6 PBS, *n*= 6 NSC-sEV, *n* = 6 MN-sEV with 3 males and 3 females in each group), and 12 for sEV and Tau oligomers (TauO) (*n =* 4 artificial cerebrospinal fluid (aCSF; whose composition is: 124 mM sodium chloride (NaCl), 2.5 mM potassium chloride (KCl), 1.2 mM sodium phosphate monobasic (NaH_2_PO_4_), 24 mM sodium bicarbonate (NaHCO_3_), 5 mM 4-(2-hydroxyethyl)-1-piperazineethanesulfonic acid (HEPES), 13 mM glucose, 2 mM calcium chloride dihydrate (CaCl_2_ ·2H_2_O), and 1.2 mM magnesium sulfate heptahydrate (MgSO_4_ 7H_2_O), at pH 7.3–7.4), *n* = 4 NSC-sEV + TauO, *n* = 4 MN-sEV + TauO). Male mice were primarily used due to concerns regarding the role of steroid sex hormones in reducing microglial inflammatory potential (Colonna and Butovsky, 2017). All animals were housed under controlled conditions with a 12-hour light/dark cycle, at 22 ± 3°C and 60% ± 5% humidity. The mice were acclimated for at least 1 week before the experiments, housed four or five per cage and given *ad libitum* access to food and water. All animal procedures were performed in compliance with the Institutional Animal Care and Use Committee at the University of Texas Medical Branch (approval No. 0910062E, approval date October 1, 2024), following ARRIVE guidelines and recommendations in the Guide for the Care and Use of Laboratory Animals of the National Institutes of Health. Mice were euthanized 24 hours after sEV administration (48 hours for sEV + Tau-treated groups) using the “drop method.” Briefly, **~**500 μL of isoflurane (NDC66794-017-10, Piramal Critical Care, Inc., Bethlehem, PA, USA) was applied to a 4 cm × 4 cm gauze pad using a 1-mL syringe, and the gauze was placed in the bottom of a desiccator. After 5 min, once sufficient vapor had accumulated, the mouse was placed on the perforated platform and the chamber sealed. Approximately 1 minute after respiration ceased and the animal no longer responded to toe pinch, it was removed, and eutahansia was confirmed. The chest cavity was then opened, and mice were transcardially perfused with 1× PBS to preserve brain tissue. Following complete perfusion, animals were decapitated, and brains were collected for immunofluorescence or molecular analyses. The brains were removed and processed for immunostaining (see below), or further dissected, snap frozen on dry ice and stored at − 80°C.

### Neural stem cell and mature neuron cultures

Adult rat hippocampus-derived NSC (Cat# SCR022) were purchased from MilliporeSigma (Tenecula, CA, USA). NSC were cultured as neurospheres in low-attachment plates in expansion media consisting of EmbryoMax DMEM/F12 with L-Glutamine, without 2-(2-hydroxyethyl)piperazin-1-yl)ethanesulfonic acid (HEPES) (MilliporeSigma, Cat# DF-042-B) B27-with retinoic acid (Gibco, Thermo Fisher Scientific, Waltham, MA, USA, Cat# 12587010), GlutaMAX^TM^ (Gibco, Thermo Fisher Scientific, Cat# 35050061), FGF-β (20 ng/mL, MilliporeSigma, Cat# GF003) and antibiotics (PSF; Gibco, Thermo Fisher Scientific, Cat# A1113803). Cells were passaged every 5–7 days using Neuropapain (Genlantis, San Diego, CA, USA, Cat# AMS.NM100200). For the generation of MN, NSC neurospheres were dissociated using Neuropapain, 2 mg/mL in basal media, plated out onto poly-ornithine/laminin-coated plates at a density of 8.0 × 10^5^ cells/cm^2^, and cultured in differentiation media consisting of expansion media without FGF-β and with the addition of 1 μM retinoic acid (MilliporeSigma, Cat# 554720) and 5 μM forksolin (MilliporeSigma, Cat# 344282) for 5 days.

### Small extracellular vesicle isolation and characterization

sEV were isolated from conditioned culture media using the ultracentrifugation method (Micci et al., 2019; Krishnan et al., 2025). Briefly, 225 mL of conditioned media collected from approximately 60 million NSC or MN cells were centrifuged at 2000 × *g*, at 4°C for 10 minutes to remove cells and debris. The resulting supernatant was centrifuged at 10,000 × *g*, at 4°C for 10 minutes, transferred to Beckman 60 Ti ultracentrifuge tubes and centrifuged at 126,000 × *g*, for 3 hours at 4°C. The resulting pellet was resuspended in 2 mL PBS containing a protease Halt Protease Inhibitor Cocktail cocktail (Cat# 78425, ThermoFisher Scientific), and centrifuged at 135,000 × *g*, for 1 hour at 4°C in a Beckman TLA110 centrifuge (Beckman Coulter, Indianapolis, IN, USA). The resulting pellet, containing an enriched fraction of sEV, was resuspended in 1 × PBS containing protease inhibitors to a concentration of approximately 1 × 10^9^ sEV/µL.

Comprehensive characterization of sEV preparation included electron microscopy (EM), western blotting (WB), and nanoparticle tracking analyses. For EM, approximately 5 μL of sEV preparation was incubated on carbon film grid (200 mesh, copper), washed, and stained with uranyl acetate. Images were acquired on a Philips CM-100 transmission electron microscope at 60 kV with an Orius SC2001 digital camera (Gatan, Pleasanton, CA, USA). WB analysis was conducted using 20 μL of sEV preparation lysed in Radioimmunoprecipitation Assay (RIPA) buffer (for a 2× solution: 75 mM sodium chloride (NaCl), 25 mM sodium phosphate dibasic (Na_2_HPO_4_), 1 mM Ethylenediaminetetraacetic acid (EDTA), 0.5% (v/v) NP-40, 0.5% TritonX-100) supplemented with protease inhibitors. Protein separation was achieved via sodium dodecyl sulfate–polyacrylamide gel electrophoresis (ThermoFisher Scientific, Cat# NP0341BOX), followed by transfer onto a nitrocellulose membrane (Global Life Sciences Solutions USA LLC, Wilmington, DE, USA, Cat# 10600011). The membrane was incubated overnight at 4°C with primary antibodies targeting CD9 antigen (CD9), CD63 antigen (CD63), CD81 antigen (CD81), Heat shock 70 kDa (Hsp70), and 130 kDa cis-Golgi matrix protein (GM130) (SBI Biotechnology, Palo Alto, CA, USA, Cat# EXOAB-KIT-1). Detection and quantification were performed using IR Dye® 680 nm and 800 nm secondary antibodies (LI-COR Biosciences, Lincoln, NE, USA, Cat# 926-68071 and 926-32211). Nanoparticle tracking analysis was performed using the NanoSight N300 system and NTA 2.1 operating system (Malvern Panalytical Inc., Westborough, MA, USA) according to the manufacturer’s instructions. Briefly, the sEV preparation was diluted in PBS and injected into the NanoSight for analysis of both particle size and concentration.

### Tau oligomers preparation and characterization

Recombinant TauOs were prepared by Dr. Rakez Kayed’s laboratory following established protocols (Gerson et al., 2017; Sengupta et al., 2018). Briefly, Tau monomers (1 mg/mL) were diluted in 1× PBS to a final concentration of 0.3 mg/mL. To initiate oligomerization, 7 μL of Aβ42 or α-synuclein oligomers (0.3 mg/mL) were added to the Tau monomer solution. The mixture was gently pipetted for 1 minute to ensure homogeneity, followed by incubation at room temperature for 1 hour on an orbital shaker. Following incubation, TauO were purified using a TSK-GEL G3000 SWXL size-exclusion chromatography column. The mobile phase consisted of 1× PBS (pH 7.4) with a flow rate of 0.5 mL/min. The eluted TauO were collected and subsequently used to seed additional Tau monomer solutions. This seeding process was repeated three times to amplify TauO formation. To characterize the presence of TauO, WB analysis was conducted: briefly, 0.5–1 μg of TauO preparation and 15 μg of total protein isolated from human AD brain homogenate and lysed in Radioimmunoprecipitation Assay buffer supplemented with protease inhibitors were used. Protein separation was achieved via sodium dodecyl sulfate–polyacrylamide gel electrophoresis (Cat# NP0341BOX, ThermoFisher Scientific), followed by transfer onto a nitrocellulose membrane (Global Life Sciences Solutions USA LLC). The membrane was incubated overnight at 4°C with primary antibody targeting Tau (mouse anti-Tau 5, Biolegend, San Diego, CA, USA, Cat# 806401, RRID: AB_2564704), whose epitope lies between amino acids 210-230 of human Tau. Detection was performed using IR Dye® 800CW goat anti-mouse IgG secondary antibody (LI-COR Biosciences, Cat# 926-32210) incubated for 1 hour at room temperature. Quantification analysis was not performed because the purpose of western blotting was purely qualitative.

### Small extracellular vesicle labeling

sEV were labeled using the PKH26 Red Fluorescent Cell Linker Kit (Cat# PKH26GL-1KT, Sigma-Aldrich, St. Louis, MO, USA). 2 μM PKH26 were added to the sEV suspension and incubated for 4 minutes under sterile conditions. After the addition of 1% bovine serum albumin (BSA)/PBS, the sEV were centrifuged at 135,000 × *g* for 1 hour at 4°C, resuspended in PBS, and stored at 4°C.

### Intracerebroventricular injections

#### Small extracellular vesicles

Mice were anesthetized with an isoflurane (Piramal Critical Care) /oxygen gas mixture (3%–5% isoflurane for induction, maintained at 1%–3% for the duration of the procedure). Intracerebroventricular (ICV) injections, on the right hemisphere, were performed using the freehand injection method, which does not require stereotaxic fixation or prior preparation of the skull (Dineley et al., 2010). This method ensures delivery and distribution of sEV throughout the brain (McCosh and Young, 2024). It is relevant to note that the hippocampus – the region on which our investigations were focused – is considered the floor of the lateral ventricles (Stratchko et al., 2016), therefore, it may be considered one of the primary targets upon ICV delivery. The site of injection was located approximately 0.5 cm from the line connecting the lateral margins of the eye sockets, **~**0.46 mm posterior to bregma, and 1 mm lateral to the midsagittal plane (Paxinos and Franklin, 2001). A 29-gauge needle, secured with hemostatic forceps to leave 4.5 mm of the needle tip exposed, was connected to a 25 μL Hamilton syringe via 0.38 mm polyethylene tubing. Infusions, for both sEV and PBS, were performed at the rate of 3 μL/min for a total volume of 3 μL, using an electronic programmable microinfuser (Harvard Apparatus, Holliston, MA, USA). Following injection, the needle was left in place for 1 minute prior to removal, after which mice were allowed to recover on a heated pad under warm light.

#### Tau oligomers

Mice were anesthetized with isoflurane (as described above) and subjected to ICV injections of either sEV or PBS as described above. 24 hours after mice were injected with 3 µL of 0.55 mM TauOs or aCSF (vehicle for TauO). The TauO concentration was chosen based on previous studies demonstrating cellular and molecular alterations relevant to Tau pathology with concentrations ranging from low nanomolar to low micromolar (Hill et al., 2019; Marcatti et al., 2024; Balducci et al., 2025; Krishnan et al., 2025)

### Free-floating immunofluorescence and small extracellular vesicles tracking

Mice brains were quickly removed, post-fixed with 4% paraformaldehyde (PFA) for 48 hours at 4°C, then transferred to 1× PBS + 0.01% sodium azide at 4°C and shipped to Neuroscience Associates (Knoxville, TN, USA) for tissue processing, using patented MultiBrain® technology. Briefly, the mouse brains were embedded together in a gelatinous block and freeze-sectioned at 35-µm in the coronal plane through the cerebrum. All sections were collected into a series of 24 cups containing Antigen Preserve solution. Free-floating immunofluorescence (FrFl-IF) was performed as previously described (Saieva and Taglialatela, 2022) with minor modifications. Briefly, non-specific binding sites were blocked with 5% BSA (Cat# A4503-100G, Sigma-Aldrich)/10% normal goat serum (NGS, Vector Laboratories, Burlingame, CA, USA, Cat# S-1000) in 1× Tris-buffered saline (TBS), and the sections were permeabilized with 0.5% Triton X-100 and 0.05% Tween-20 for 1 hour at room temperature. The slices were then incubated with the following primary antibodies containing 1.5% NGS in 1×TBS, overnight at 4°C: rabbit anti-ionized calcium-binding adapter-1 (Iba1; 1:200, Wako, Fujifilm Wako Pure Chemicals Corporation, Osaka, Japan, Cat# 019-19741, RRID: AB_839504), chicken anti-glial fibrillary acidic protein (GFAP; 1:500, Aves Labs, Davis, CA, USA, Cat# GFAP, RRID: AB_2313547), rabbit anti-neuronal nuclei antigen (NeuN; 1:500, Cell Signaling, Danvers, MA, USA, Cat# 12943, RRID: AB_2630395), rat anti-cluster of differentiation 68 (CD68, also known as macrosialin, 1:500, Abcam, Cambridge, MA, USA, Cat# ab53444, RRID: AB_869007). After washing with 1× TBS, the slices were incubated for 1 hour at room temperature with the Alexa-conjugated secondary antibodies (goat anti-rabbit Alexa Fluor 488, 1:400, Invitrogen, Carlsbad, CA, USA, Cat# A-11008, RRID: AB_143165; goat anti-chicken Alexa Fluor 488, 1:400, Thermo Fisher Scientific, Cat# A-11039, RRID: AB_2534096; goat anti-rat Alexa Fluor 647, 1:400, Thermo Fisher Scientific, Cat# A48265, RRID: AB_2895299) in 1× TBS containing 1.5% NGS. After washing with 1 × TBS, the slices were adhered onto glass slides (50 mm × 76 mm × 1.2 mm, Neuroscience Associates, Cat# 10004) and air-dried. Finally, the slices were treated with the anti-autofluorescence kit TrueView (Cat# SP-8400, VectorLab, Newark, CA, USA) for 2 minutes, briefly washed in 1× TBS, coverslipped using Fluoromount-G containing 4′,6-diamidino-2-phenylindole (DAPI; SouthernBiotech, Birmingham, AL, USA, Cat# 0100-20), and sealed.

### On-slide immunofluorescence

Collected brains were post-fixed in 4% paraformaldehyde for 48 hours at 4°C, then cryoprotected by suspension in 30% sucrose solution for 48 hours at 4°C. Brains were then embedded in optical cutting temperature (OCT) compound (Tissue-Tek, Tokyo, Japan) and stored at –80°C. For on-slide immunofluorescence (IF) experiments, mouse brain sections of 12 µm were cut using a cryostat (Microm HM 525, Thermo Fisher Scientific) and collected onto Super Frost slides (Thermo Fisher Scientific, Cat# 12-550-15). Slides were post-fixed in 4% paraformaldehyde for 30 minutes. After three washes in 1× PBS, pH 7.4, non-specific binding sites were blocked with 5% BSA (Sigma-Aldrich)/10% NGS (Thermo Fisher Scientific) and sections were permeabilized with 0.5% Triton X-100/0.05% Tween-20 for 1 hour at room temperature (21°C). Incubation with primary antibodies in 1× PBS containing 1.5% NGS was carried out overnight at 4°C (chicken anti-Tau 1:100, Abcam, Cat# ab75714, RRID: AB_1310734; rabbit anti-Iba1 1:200, FUJIFILM Wako, Cat# 19741). Slides were then washed three times in 1× PBS and incubated for 1 hour at room temperature with appropriate secondary antibodies diluted in 1× PBS containing 1.5% NGS (goat anti-chicken Alexa Fluor 594, 1:400, Thermo Fisher Scientific, Cat# A-11042, RRID: AB_2534099; goat anti-rabbit Alexa Fluor 647, 1:400, Thermo Fisher Scientific, Cat# A32733, RRID: AB_2633282). Slides were again washed three times in 1× PBS, rinsed in 70% ethanol and then incubated for 10 minutes at room temperature in 70% ethanol containing 0.3% Sudan Black to remove lipofuscin autofluorescence. Slides were briefly washed in distilled water before mounting with Fluoromount-G containing DAPI and sealed.

### Microscopy, image analyses, and quantification

All images were acquired with a Keyence BZ-X800 (Keyence Corporation, Osaka, Japan) microscope using immersion oil 60× or 100× objective. For FrFl-IF two consecutive sections were analyzed, and three to four images from each hippocampal subregion (for a total of 9–12 images from each analyzed area: cornu ammonis 3, CA3, cornu ammonis 1, CA1, and DG). For immunofluorescence (IF) three sections for each animal were analyzed, and at least two images per section were taken from the 3 hippocampal subregions mentioned earlier; for both FrFl and IF the images were taken at 1920 × 1440-pixel resolution, with the z-step size of 2 μm at either 12 μm (IF) or 35 µm thickness (FrFl-IF). For the feasibility of the quantification, all layers from a single image stack were projected on a single slice (stack/Z projection). Quantitative analysis was performed using ImageJ software version 1.54 (https://imagej.nih.gov/ij, National Institutes of Health, Bethesda, MD, USA) by analyzing the fluorescence intensity of each marker (Integrated Density, IntDen, and Area). The marker expression was quantified by measuring the average fluorescence intensity, and the average percentage of stained areas of each analyzed region. A threshold for positive staining was determined for each image that included all cell bodies and processes but excluded background staining. For the sEV co-localization, the total number of cells positive for a specific marker (total marker^+^) was first determined, then a manual count for cells both positive for a specific marker and for the PKH26 labeling (marker^+^/sEV^+^) ensued. The results were expressed as percentage of cells double positive over the total number of cells positive for a specific marker (% marker^+^sEV^+^/marker^+^). The colocalization between Tau and Iba1 was evaluated and quantified using Pearson’s correlation coefficient analysis (PCC). Representative images were composed in CC2024 format in Adobe Photoshop (San Jose, CA, USA).

### Bulk RNA-sequencing

Mice were euthanized as described earlier. After brain extraction, the hippocampal tissues were collected for bulk RNA-sequencing (RNA-seq) analysis, and two distinct tissue preparations were obtained: total homogenate and synaptosome fraction. The total homogenate was prepared according to previous publications (Zolochevska and Taglialatela, 2020): briefly, the tissue was placed in Trizol and homogenized using the Polytron homogenizer (Thermo Fisher Scientific). Chloroform was then added, and the samples were centrifuged at 15,000 × *g*, for 15 minutes at 4°C. The aqueous phase was transferred to a new tube containing isopropanol. A further centrifugation of the samples was performed at 15,000 × *g*, for 10 minutes at 4°C. The resulting pellet was collected, then washed with ice-cold 80% ethanol and air-dried. The samples were resuspended in 40 μL nuclease-free water. The RNA concentration was measured using NanoDrop 2000c (Thermo Fisher Scientific). The synaptosome fraction was prepared as previously described (Marcatti et al., 2022), using Syn-PER Reagent (Thermo Fisher Scientific). Briefly, 30 mg of tissue was homogenized using Dounce glass homogenizer in the presence of protease inhibitors (Thermo Fisher Scientific) and Phosphatase Inhibitor Cocktail (MilliporeSigma, Cat# 5246251SET). The homogenate was centrifuged at 1200 × *g*, for 10 minutes at 4°C. The supernatant was collected and spun down at 15,000 × *g*, for 20 minutes at 4°C. The resulting pellet contains synaptosomes. The pellet was then resuspended in HBK (HEPES-buffered Krebs-like) buffer and stored at − 80°C.

### Single-nuclei extraction

Twenty-five milligrams of hippocampal tissue were utilized for single-nuclei extraction. The isolation was performed using the Chromium Nuclei Isolation Kit with RNase Inhibitor (PN-1000494, 10x Genomics, Pleasanton, CA, USA), following the user guide. Briefly, the hippocampal tissue was pre-chilled in dry ice inside the Sample Dissociation Tubes provided with the kit. Each sample received 200 µL of Lysis Buffer (proprietary composition). Tissue dissociation was achieved using a pestle in an ice-cold environment, followed by further addition of 300 µL of lysis buffer, and incubation for 10 minutes on ice. The lysates were then transferred to pre-chilled Nuclei Isolation Columns and centrifuged at 20,000 × *g* for 20 seconds. After careful removal of the supernatant, the resulting pellet was mixed with Debris Removal Buffer (proprietary composition) and spun down, with the supernatant being discarded. The nuclei pellet was washed twice with wash and resuspension buffer (10% BSA in 1× PBS with RNAse inhibitor provided with the Nuclei Isolation kit). Finally, the pellet was resuspended in the same buffer and kept on ice for immediate library construction. To determine the nuclei concentration, an aliquot of the nuclei suspension was mixed with an equal volume of Trypan Blue and manually counted using a hematocytometer under an inverted microscope.

### Library construction

After successful nuclei isolation, the library construction was performed using the Chromium Next GEM Single Cell 3’ Reagent Kits v3.1 (Dual Index) (the reagent kit, Cat# PN-1000268 and PN-1000269; the chips, PN-10000120 and PN-1000127; the Dual Index Kit barcodes, PN-1000215, 10x Genomics) according to the manufacturer’s user guide. Briefly, the nuclei underwent barcoding after mixing them with gel beads and partitioning oil onto a Chromium Next Gem Chip G, with a target recovery of 10,000 nuclei per sample. The chip was processed in a Chromium Controller (10x Genomics) to generate Gel Beads-in-emulsion (GEMs). Following reverse transcription using a GEM–reverse transcription (RT) incubation protocol on a polymerase chain reaction (PCR) cycler, GEMs were broken using Recovery Agent (10x Genomics). The resulting cDNA was purified with DynaBeads MyOne Silane beads (10x Genomics, Cat# PN-2000048), followed by amplification with PCR for 11 cycles. Further purification was achieved using SPRIselect magnetic beads (Beckman Coulter, Cat# B23318). The PCR products were subjected to quality controls and quantified using a TapeStation 4200 using the Agilent High Sensitivity D5000 ScreenTape System (Agilent, Santa Clara, CA, USA). The snRNA-seq libraries were constructed utilizing 25% of the cDNA yield (10 µL). Following fragmentation, end repair, A-tailing, and size selection purification, the cDNA underwent ligation with adaptors and subsequent purification using SPRISselect beads. For library amplification, PCR was performed with sample index oligonucleotides from the Chromium i7 Multiplex Kit (10x Genomics), with a total of 10 or 11 PCR cycles, estimated based on the initial cDNA input. To ensure optimal library size distribution, final PCR products underwent double-sided size selection using SPRIselect beads at ratios of 0.6× and 0.8×. Library quality control was conducted on Agilent TapeStation 4200. Sequencing was performed at a depth of 20,000 paired reads per cell on an Illumina NovaSeq 550 sequencer with the following (PE150) sequencing settings: Read 1, 28 cycles; i7 index, 8 cycles; Read 2, 91 cycles.

### Preprocessing, noise removal, and cell-type identification of RNA datasets

Fastq files were uploaded onto the 10x Genomics Cloud and were aligned to the mouse genome (mm10) reference transcriptome, then preprocessed in Cell Ranger v7.0.0 (10x Genomics). The single-cell RNA-seq dataset was processed using the CellBender bioinformatic algorithm (Fleming et al., 2023), thanks to Biomage, Inc. (open-source company registered in Scotland, UK, now acquired by Parse Biosciences, Seattle, WA, USA, https://app.trailmaker.parsebiosciences.com/) with the goal of removing ambient RNA that might interfere with the analyses. The CellBender algorithm was used with the following parameters: false positive rate, 0.01, expected “cells” ranging from 1500 to 3800, total droplets included 10,000, epochs 150, then explored and visualized using Cellenics® community instance (now Trailmaker™) originally developed by Biomage. The following filters were used: mitochondrial content filter automatically set at 0.3; number of genes vs number of Unique Molecular Identifier, UMI filter automatically set at 0.03; doublet filter automatically set at 0.02. Normalization was performed using a linear regression method, while the data were integrated with Harmony and the number of top high variable genes (#HVGs) was set at 2000. Dimensionality reduction was performed using principal component analysis and Uniform Manifold Approximation and Projection (UMAP). Clustering was executed using the Louvain algorithm; annotation and identification of cell types were conducted using ScType, a biocomputational method based on specific marker combinations (Ianevski et al., 2022; Nader et al., 2024), implemented within Cellenics.

### Differentially expressed genes, Gene Ontology, and pathway enrichment analysis

After cluster identification and annotation, a list of differentially expressed genes (DEGs) was created through Cellenics and downloaded; we then selected the up-regulated and down-regulated genes in NSC-sEV vs. MN-sEV groups; we set as cutoff a Log Fold Change (LFC) ≥ 1 or ≤ –1 for the genes resulting up-regulated or down-regulated, respectively, and a *P*-value < 0.05.

After obtaining the DEGs list, we interrogated the databases EnrichR (web-based enrichment analysis platform, available online at https://maayanlab.cloud/Enrichr/) (Chen et al., 2013; Kuleshov et al., 2016; Xie et al., 2021) and PantherDB (Protein ANalysis THrough Evolutionary Relationships; web-based database for functional and pathway enrichment analysis; http://www.pantherdb.org), through Cellenics, to generate a list of enriched pathways, biological processes, cell components, molecular functions, protein class detected; namely Gene Ontology terms (GO terms) (Mocciaro et al., 2023).

### Data analysis

Microscope images analysis: The results of markers (Iba1 and CD68) integrated density, sEV co-localization with specific cell markers (% marker^+^sEV^+^/marker^+^), and Pearson correlation coefficient between TauO and Iba1 were statistically analyzed. The statistical analyses were conducted using GraphPad Prism 9.3.1 software (GraphPad Inc., San Diego, CA, USA). Significant differences between groups were determined with one-way analysis of variance followed by Tukey’s *post hoc* tests. Data are expressed as mean ± SD, and *P* < 0.05 was considered statistically significant for all analyses.

Bulk-RNA sequencing: Differences in messenger RNA levels between the experimental groups were screened by performing multiple comparison tests of gene expression levels, applying a False Discovery Rate (FDR) *P*-value for the practical difference test. The Benjamini-Hochberg procedure was used to control the FDR. The ‘Practical Dif PValue’, was used to determine the statistical significance of the mean differences in gene responses across groups. A small *P*-value in this context signified a substantial difference, exceeding the pre-set significance threshold. DEG underwent pathway enrichment analysis using GO terms. DEG analysis was conducted using JMP software and gene enrichment pathway analysis was performed with Metascape (http://metascape.org) (Zhou et al., 2019).

## Results

### Isolation and characterization of small extracellular vesicles

In this study, we utilized an enriched fraction of sEV derived from adult rat hippocampal NSC and from MN, which was obtained after differentiation of NSC. sEV from both cell types were isolated from the culture media and characterized according to the guidelines of the International Society of Extracellular Vesicles (Lötvall et al., 2014), and as previously reported (Micci et al., 2019; Krishnan et al., 2025) by EM analysis, WB analysis of specific markers, and single particle tracking analysis (**Additional Figure 1**). EM (**Additional Figure 1A**) revealed the presence of round particles with a double membrane, and single particle tracking analysis using the NanoSight system (Malvern Inc.) confirmed the size distribution profile indicative of a physically homogeneous population, with a peak in the range between **~**60–150 nm for both sEV types (**Additional Figure 1B**). Finally, WB analyses (**Additional Figure 1C**) confirmed that the preparation is enriched in sEV, highlighting the presence of vesicular markers (CD63, CD9, CD81, and Hsp70) and the minimal presence of the Golgi-associated protein GM130, which serves as a control for contamination.

### Tau oligomers characterization

We compared our TauO preparation with brain homogenate obtained from human AD homogenates (**Additional Figure 2**). After incubation with primary (Tau5) and secondary antibodies, we detected oligomers bands at around 250 and 150 kDa in all the lanes, which correspond to TauO. Therefore, we conclude that our preparation contains TauO.

### Evaluation of small extracellular vesicle uptake in the hippocampus

We assessed whether sEV uptake was different among neurons, microglia, and astrocytes in the hippocampus of mice treated with NSC-sEV and MN-sEV conjugated with a red fluorescent dye, PKH26. We evaluated the co-localization levels of PKH26-sEV with NeuN (marker of neurons), Iba1 (marker of microglia), and GFAP (marker of astrocytes), and analyzed each hippocampal area (CA1, CA3, and DG) separately.

**Figure 1:**
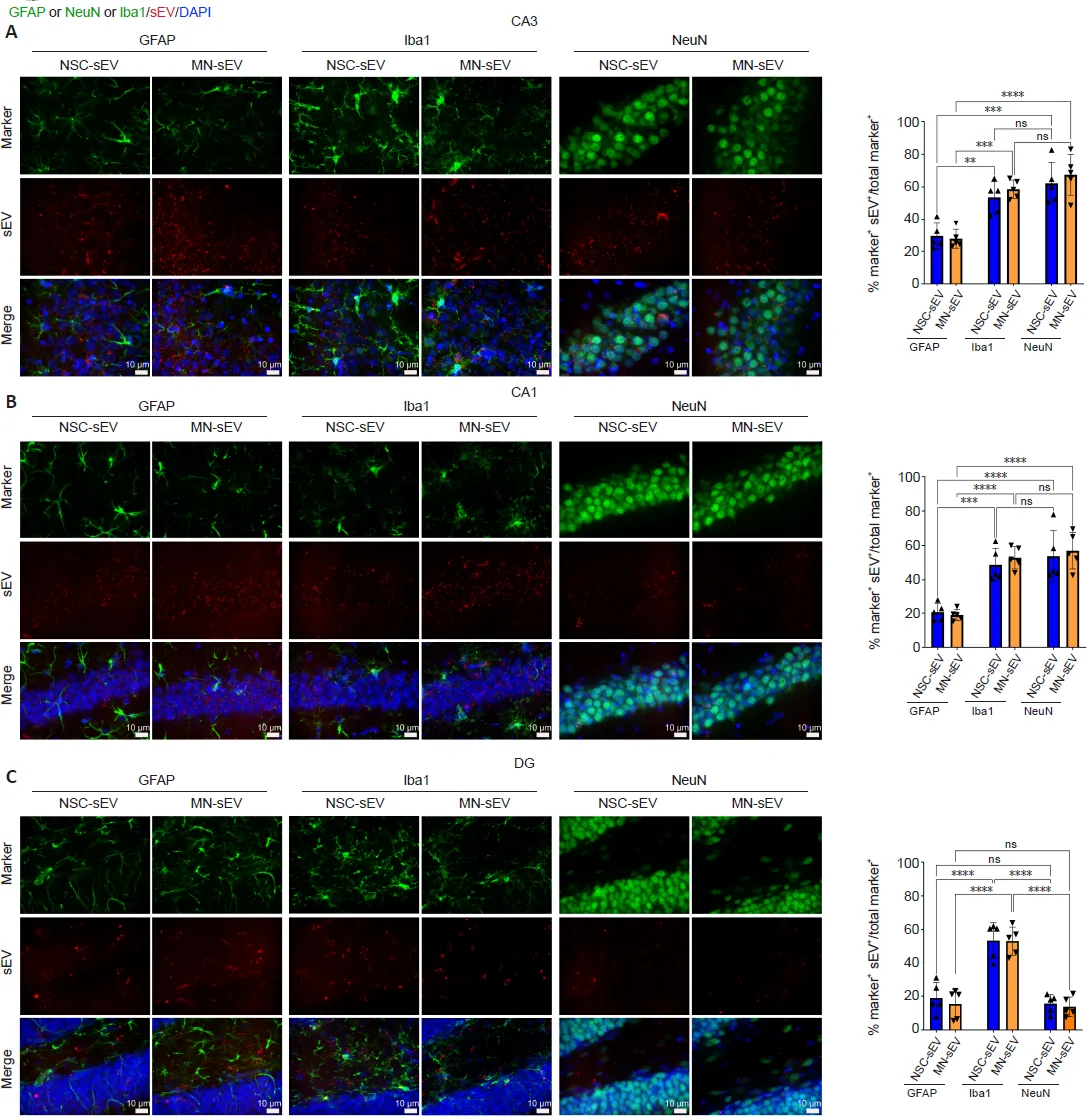
Although NSC-sEV and MN-sEV show similar uptake, microglia are the primary recipients across the hippocampus, as shown in representative images (cell markers in green), along with quantifications for both groups. The small extracellular vesicles (in red) were labeled with a fluorescent dye (PKH26) prior to their intracerebroventricular injection in wild-type male mice. 24 hours following the injections, sEV’s uptake and co-localization with different cell markers were assessed, in the three different hippocampal regions CA3 (A) and CA1 (B), and DG (C), revealing preferential uptake of sEV by microglia. Scale bars: 10 µm. Data are presented as mean ± SD. **P < 0.01, ***P < 0.001, ****P < 0.0001. CA1: Cornu ammonis 1; CA3: cornu ammonis 3; DAPI: 4′,6-diamidino-2-phenylindole; DG: dentate gyrus; GFAP: glial fibrillary acidic protein; Iba1: ionized calcium-binding adapter-1; MN-sEV: mature neuron-derived small extracellular vesicles; NeuN: neuronal nuclei; ns: not significant; NSC-sEV: neuronal stem cell-derived small extracellular vesicles.

In all analyzed areas, we found no significant differences between NSC-sEV and MN-sEV uptake for each examined cell type (**Table 1** and **Figure 1**). However, we also found that sEV were preferentially taken up by microglia and neurons in the CA3 (**Table 2** and **Figure 1A**) and CA1 regions (**Table 2** and **Figure 1B**), and by microglia in the DG (**Table 2** and **Figure 1C**).

**Table 1:** Statistical comparison of NSC-sEV and MN-sEV uptake in different cell types across hippocampal regions

Cell type	CA3	CA1	DG
Astrocytes (GFAP)	0.7044	0.543	0.1755
Microglia (Iba1)	0.3429	0.4506	0.9612
Neurons (NeuN)	0.529	0.7073	0.6059

The table summarizes the statistical comparisons (*P*-values) of NSC-sEV versus MN-sEV uptake across different cell types in distinct hippocampal areas. Statistical analysis: two-tailed parametric unpaired t-test. # “marker” refers to the specific cell identifier used to mark the different cell types; sEV uptake (NSC-sEV vs. MN-sEV) in different cell types across hippocampal regions (% marker^+^/sEV^+^)^#^. CA1: Cornu ammonis 1; CA3: cornu ammonis 3; DG: dentate gyrus. GFAP: glial fibrillary acidic protein; Iba1: ionized calcium-binding adapter-1; MN-sEV: mature neuron-derived small extracellular vesicles; NeuN: neuronal nuclei; NSC-sEV: neuronal stem cell-derived small extracellular vesicles.

**Table 2:** Statistical comparison of NSC-sEV and MN-sEV co-localization with distinct cell markers across hippocampal regions

	CA3			CA1			DG	
				
	NSC-sEV	MN-sEV		NSC-sEV	MN-sEV		NSC-sEV	MN-sEV
	One-way analysis of variance with Tukey’s *post hoc* test							
	0.0008^***^	< 0.0001^****^		0.0007^***^	< 0.0001^****^		< 0.0001^****^	< 0.0001^****^
Astrocytes *vs*. Microglia	0.0077^**^	0.0003^***^		0.0035^**^	< 0.0001^****^		0.0001^***^	< 0.0001^****^
Astrocytes *vs*. Neurons	0.0008^***^	< 0.0001^****^		0.0008^***^	< 0.0001^****^		0.8191	0.9402
Microglia *vs*. Neurons	0.4096	0.2721		0.7433	0.6677		< 0.0001^****^	< 0.0001^****^

In this table it is shown the statistical comparison (*P*-values) of NSC-sEV and MN-sEV co-localization with distinct cell markers of astrocytes, microglia, and neurons. ^**^P < 0.01, ^***^P < 0.001, ^****^P < 0.0001. # “marker” refers to the specific cell identifier used to mark the different cell types; co-localization of sEV and specific markers across hippocampus ([% marker^+^/sEV^+^]/total marker^+^)^#^. CA1: cornu ammonis 1; CA3: cornu ammonis 3; DG: dentate gyrus; MN-sEV: mature neuron-derived small extracellular vesicles; NSC-sEV: neuronal stem cell-derived small extracellular vesicles.

In summary, although we found no difference in the uptake of either type of sEV among the three cell types analyzed, microglia appear to consistently interact with the sEV throughout the hippocampus. This suggests that the immune response plays a significant role in mediating the effects of sEV in the brain.

### Effects of small Extracellular Vesicles on microglia activation in the hippocampus

To assess whether the higher uptake of sEV by hippocampal microglia leads to their activation, we measured the levels of Iba1, a marker that identifies microglia, and CD68, a marker of microglia activation.

**Figure 2:**
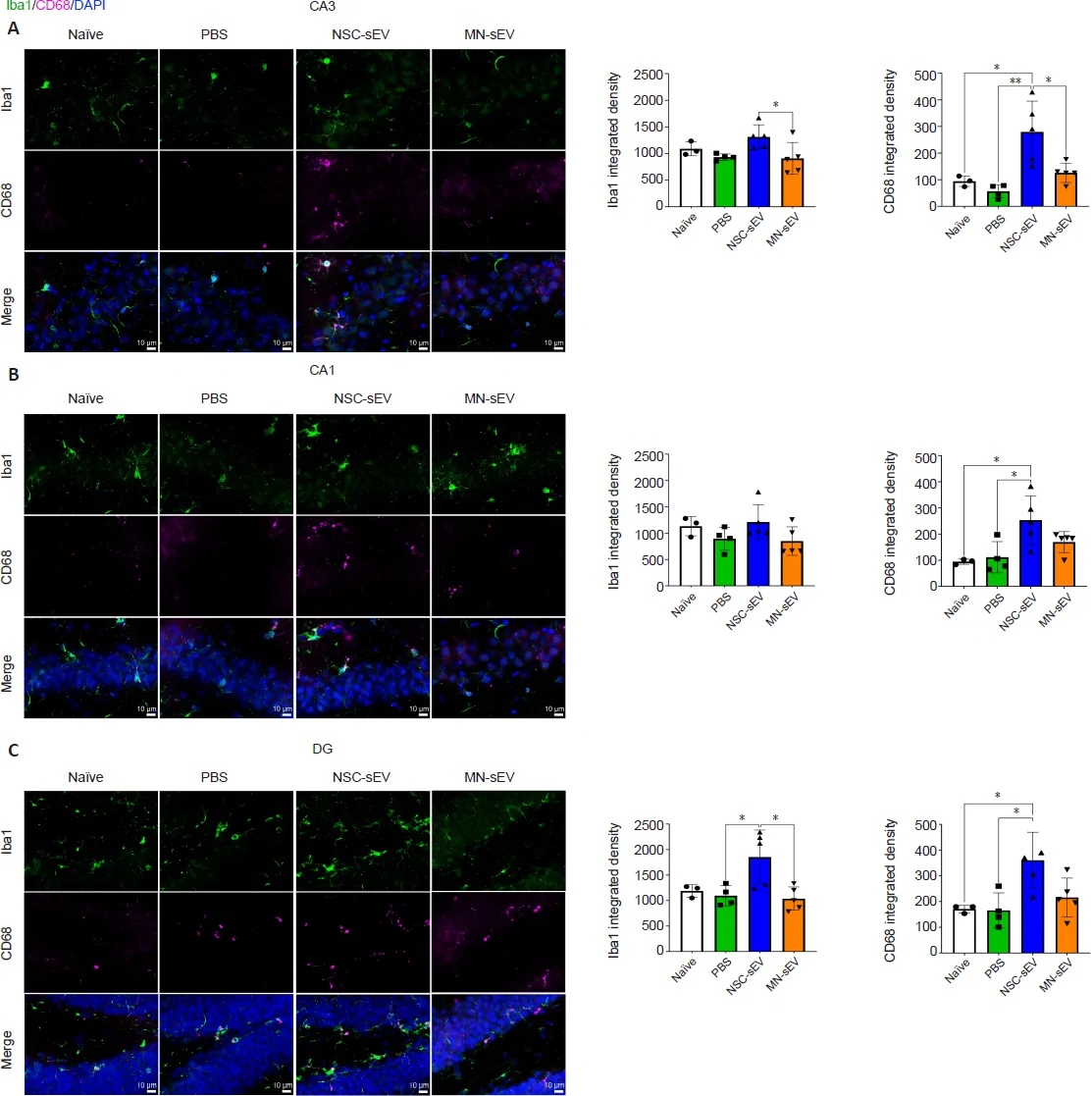
NSC-sEV increase microglial activation in the hippocampus, as shown by representative Iba1- and CD68-stained images from naïve, PBS-, NSC-sEV-, and MN-sEV-injected mice, with relative quantifications. Levels of Iba1 (total microglia) and CD68 (lysosomal marker of microglial activation) in CA3 (A), CA1 (B), and DG (C) areas of the hippocampus were compared. NSC-sEV induced microglia activation throughout the hippocampus. Scale bars: 10 µm. Data are presented as mean ± SD. *P < 0.05, **P < 0.01. CA1: Cornu ammonis 1; CA3: cornu ammonis 3; CD68: cluster of differentiation 68; DAPI: 4′,6-diamidino-2-phenylindole; DG: dentate gyrus; Iba1: ionized calcium-binding adapter-1; MN-sEV: mature neuron-derived small extracellular vesicles; NSC-sEV: neuronal stem cell-derived small extracellular vesicles.

In CA3 (**Figure 2A** and **Table 3**), we found no significant differences in Iba1 and CD68 levels between the control groups and either NSC-sEV or MN-sEV groups. However, Iba1 levels were significantly higher in NSC-sEV group compared with the MN-sEV group, suggesting a higher number of microglial cells in the former group. Additionally, we observed a significant increase in CD68 levels in the NSC-sEV group compared with the control group, as well as in the NSC-sEV group compared with the MN-sEV group. In CA1 (**Figure 2B** and **Table 3**), no significant differences were observed in Iba1 levels. In the DG (**Figure 2C** and **Table 3**), Iba1 levels were higher in the NSC-sEV group compared with the PBS and MN-sEV groups, similarly to CA1, while CD68 levels were significantly higher in the NSC-sEV group compared with the controls. Overall, these data suggest that NSC-sEV, but not MN-sEV, induce microglia activation across the hippocampus, supporting the hypothesis that the effects of NSC-sEV in the brain may be partially mediated by the immune response through microglia activation.

**Table 3:** Statistical comparison of Iba1 (microglia) and CD68 (microglia activation) integrated density in immunofluorescence analyses across hippocampal regions

	CA3			CA1			DG	
				
	Iba1	CD68		Iba1	CD68		Iba1	CD68
	One-way analysis of variance with Tukey’s *post hoc* test							
	0.0449*	0.0015**		0.1679	0.0123*		0.0084**	0.0103*
Naïve *vs*. PBS	0.7847	0.8837		0.6605	0.9844		0.9830	0.9998
Naïve *vs*. NSC-sEV	0.5097	0.0124*		0.9777	0.0205*		0.0775	0.0308*
Naïve *vs*. MN-sEV	0.6783	0.9212		0.4983	0.4020		0.9254	0.8742
PBS *vs*. NSC-sEV	0.0876	0.0016**		0.3367	0.0245*		0.0242*	0.0155*
PBS *vs*. MN-sEV	0.9984	0.4576		0.9945	0.5426		0.9937	0.7987
NSC-sEV *vs*. MN-sEV	0.0492*	0.0164*		0.1969	0.2078		0.0102*	0.0601

We show here the statistical comparison (P-values) of Iba1 and CD68 integrated density in immunofluorescence experiments in four different groups of animals (naïve not injected, injected with either PBS, NSC-sEV, or MN-sEV). *P < 0.05, **P < 0.01. CA1: Cornu ammonis 1; CA3: cornu ammonis 3; CD68: cluster of differentiation 68; DG: dentate gyrus; Iba1: ionized calcium-binding adapter-1; MN-sEV: mature neuron-derived small extracellular vesicles; NSC-sEV: neuronal stem cell-derived small extracellular vesicles; PBS: phosphate buffer saline.

### Effect of small extracellular vesicles on Tau oligomer uptake by hippocampal microglia

**Figure 3:**
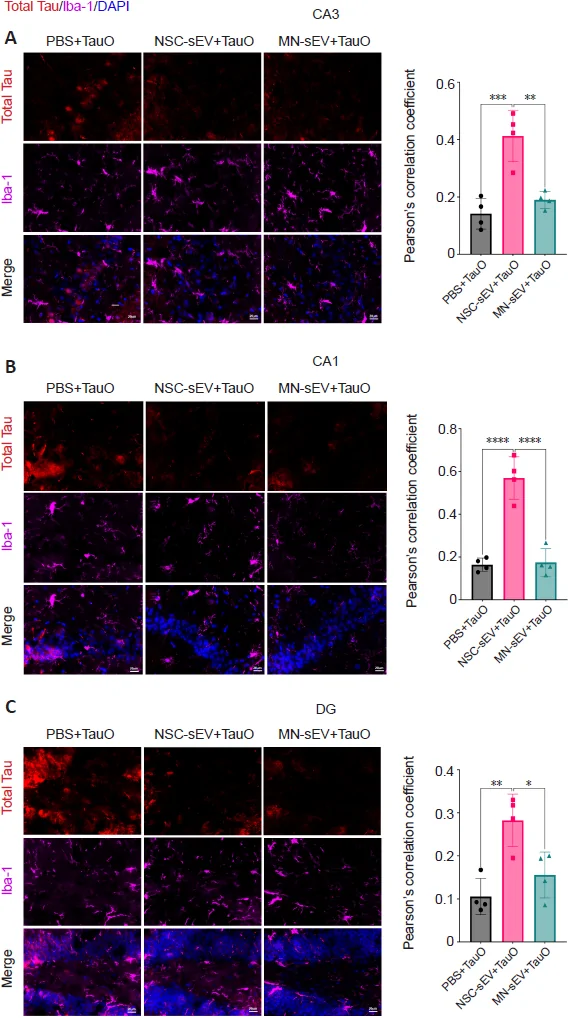
TauO uptake by microglia is increased upon NSC-sEV treatment, as shown by the colocalization analysis of Tau and Iba1 in the hippocampus of mice treated with PBS, NSC-sEV, or MN-sEV after TauO injection, and relative quantifications. Representative images and quantitative analyses of double immunofluorescence staining for Iba1 (magenta) in combination with total Tau (red) revealed a significant increase of TauO uptake in microglia after treatment with NSC-sEV in CA3, CA1, and DG. Scale bars: 20 μm. Values are expressed as the mean ± SD. *P < 0.05, **P < 0.01, ***P <0.001, ****P < 0.0001. CA1: Cornu ammonis 1; CA3: cornu ammonis 3; CD68: cluster of differentiation 68; DAPI: 4′,6-diamidino-2-phenylindole); DG: dentate gyrus; Iba1: ionized calcium-binding adapter-1; MN-sEV: mature neuron-derived small extracellular vesicles; NSC-sEV: neuronal stem cell-derived small extracellular vesicles; TauO: Tau oligomers.

To explore whether NSC-sEV-induced activation of microglia enhances the removal of toxic amyloid oligomers, known to contribute to early synaptic dysfunction in AD, we examined the uptake of ICV-injected TauO by microglial cells in the hippocampus of mice that were previously injected with NSC-sEV, MN-sEV, or PBS (**Figure 3A–C** and **Table 4**). Using IF, we determined the colocalization between Iba1 and Tau by performing a PCC analysis: mice treated with NSC-sEV have a significantly higher PCC compared with those treated with PBS or MN-sEV. This suggests that NSC-sEV-activated microglia have a greater ability to take up TauO in all the analyzed areas, thus further supporting the notion that microglia are “ready-to-act” upon NSC-sEV uptake.

**Table 4:** Statistical comparison of Pearson correlation coefficient between microglia (Iba1^+^) and Tau oligomers across hippocampal areas

	CA3	CA1	DG
	One-way analysis of variance with Tukey’s *post hoc* test		
	0.0004***	0.0001****	0.0029**
PBS+TauO *vs*. NSC-sEV+TauO	0.0005***	0.0001****	0.0027**
PBS+TauO *vs*. MN-sEV+TauO	0.5442	0.9751	0.4076
NSC-sEV+TauO *vs*. MN-sEV+TauO	0.0019**	< 0.0001****	0.0194*

Animals were first treated with either PBS or sEV, and then challenged with oligomers upon administration of either PBS or sEV. This table shows the differences (P-values) in colocalization between microglia and Tau oligomers in the different groups of animals. *P < 0.05, **P < 0.01, ***P < 0.001, ****P < 0.0001. CA1: Cornu ammonis 1; CA3: cornu ammonis 3; DG: dentate gyrus; Iba1: ionized calcium-binding adapter-1; MN-sEV: mature neuron-derived small extracellular vesicles; NSC-sEV: neuronal stem cell-derived small extracellular vesicles; PBS: phosphate buffer saline; TauO: Tau Oligomers.

### Bulk RNA-sequencing on hippocampal total homogenates and synaptosomal fractions

**Figure 4:**
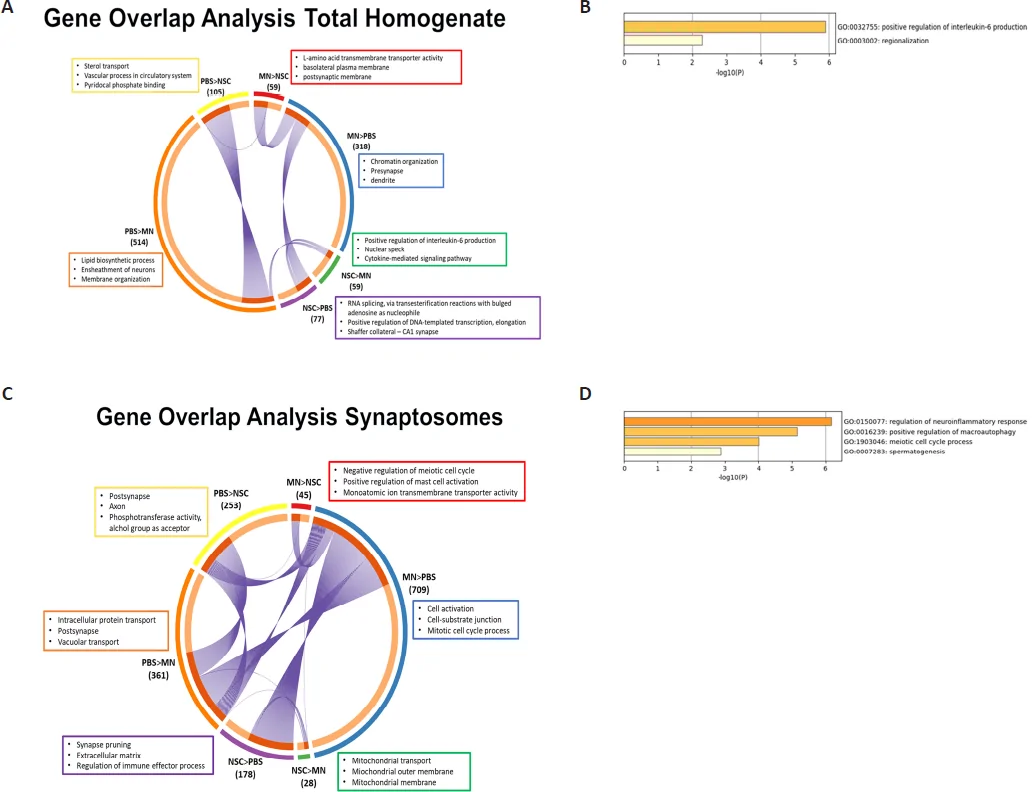
RNA-bulk sequencing shows involvement of immune response and neuroinflammation-related pathways in the NSC-sEV groups (NSC > MN). In this panel, RNA-bulk analyses of NSC-sEV, MN-sEV, and PBS DEGs groups are shown, alongside Pathway Enrichment Analyses. Circos plots integrating the number of DEGs identified using false discovery rate (FDR) when comparing two distinct groups at a time (e.g., “MN > NSC,” signifies genes that are overexpressed in mature neurons derived-sEV compared to neural stem cells derived-sEV). The purple lines that traverse the plot interconnect identical genes that appear in multiple lists. Overall differences between MN and NSC groups are inflammatory pathways in total homogenate (A), and mitochondrial pathways in synaptosome fraction (C). (B, D) The pathway enrichment analysis of DEGs between MN and NSC (excluding the ones in common with PBS) in total homogenate (A) and synaptosome fraction (B). (–log_10_ *P* represents the negative logarithmic function of *P*-value relative to the enrichment). DEGs: Differentially expressed genes; MN-sEV: mature neuron-derived small extracellular vesicles; NSC-sEV: neuronal stem cell-derived small extracellular vesicles; PBS: phosphate buffer saline.

To further investigate the impact of NSC-sEV on the hippocampus, we performed bulk RNA sequencing to identify DEGs in both tissue homogenate and synaptosomal fraction from mice injected ICV with NSC-sEV, MN-sEV, or PBS. To assess the gene response variations across different preparations, we performed a group categorization aimed at including in our comparison: NSC syn (neural stem cell synaptosome), NSC total (neural stem cell total homogenate), MN syn (MN synaptosome), and MN total (MN total homogenate), alongside PBS controls. To enhance precision, we applied adjustments for the FDR: this procedure enabled a comprehensive pathway enrichment analysis of the DEGs, and a comparison across the different groups. Analysis of the total hippocampal homogenate (**Figure 4A**) indicated that, compared with MN-sEV, administration of NSC-sEV increased the expression of genes associated with inflammatory processes, such as interleukin-6, and decreased the expression of genes related to L-amino acid transmembrane transporter activity. In the hippocampal synaptosome fraction (**Figure 4C**), NSC-sEV injection upregulated genes involved in metabolic processes, particularly those related to mitochondrial function, and downregulated genes associated with the negative regulation of the cell cycle, suggesting an influence of NSC-sEV on neurodevelopmental processes.

To further refine our analysis, we excluded DEGs common to the PBS group from those overlapping with either the NSC-sEV or MN-sEV groups to eliminate non-specific effects due to sEV treatment itself. Consistent with previous results, NSC-sEV treatment predominantly influenced neuroinflammatory pathways compared with MN-sEV treatment (**Figure 4**). A detailed analysis (**Figures 4B** and **4D**) highlighted key pathways, including interleukin-6 signaling and regionalization in the total homogenate, as well as regulation of neuroinflammation, positive regulation of macroautophagy, meiotic cell cycle process, and spermatogenesis in the synaptosome fraction.

### Single-nuclei RNA sequencing on hippocampal microglia nuclei

To investigate the effects of sEV on specific cell types, we conducted an snRNA-seq analysis on the hippocampus of mice injected ICV with NSC-sEV, MN-sEV, or PBS. Single nuclei were isolated from the hippocampi of these mice and processed into barcoded single-nuclei RNA libraries using 10x Genomics technology. We identified 21 distinct clusters (**Additional Figure 3A**), while the ScType method assigned a specific cell type to each cluster (**Additional Figure 3B**).

Based on bulk RNA sequencing results and immunostaining data described earlier, we focused specifically on the “Microglial cells” cluster (“Cluster 9”) and compared DEGs between the NSC-sEV and MN-sEV groups.

**Figure 5:**
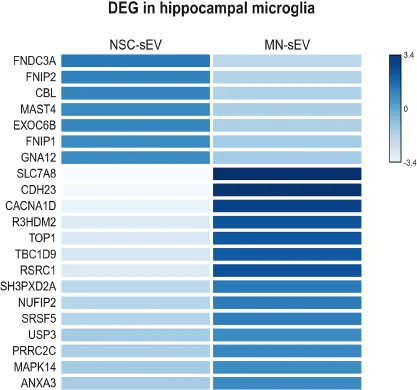
Heatmap of DEGs after single-nuclei RNA sequencing of hippocampal microglia in mice treated with NSC-sEV and MN-sEV shows differential expression of genes involved in lysosome biogenesis and microglia activation. A total of 20 genes were identified as differentially expressed between NSC-sEV and MN-sEV groups in hippocampal microglial cells. Interestingly, genes related to lysosome activity (*Fnip1* and *Fnip2*) and regulation of microglia activation (*Cbl* and *Mapk14*) were identified. On the top left, the genes upregulated in NSC-sEV-treated mice are shown, while in the bottom left, downregulated genes are shown. DEG: Differentially expressed genes; MN-sEV: mature neuron-derived small extracellular vesicles; NSC-sEV: neuronal stem cell-derived small extracellular vesicles.

We identified 20 DEGs (**Figure 5**), including *Mapk14*, involved in the p38MAPK pathway, which is activated by pro-inflammatory cytokines; *Slc7a8*, which encodes a transporter for small and large neutral amino acids; *Fnip1* and *Fnip2*, which are part of the folliculin complex involved in regulating mechanistic target of rapamycin complex 1 (mTORC1), AMPK, and lysosome biogenesis; and *Gna12*, which encodes a G-protein involved in mTOR and cytokine signaling. The full list of DEGs is presented in the heatmap (**Figure 5**).

**Figure 6:**
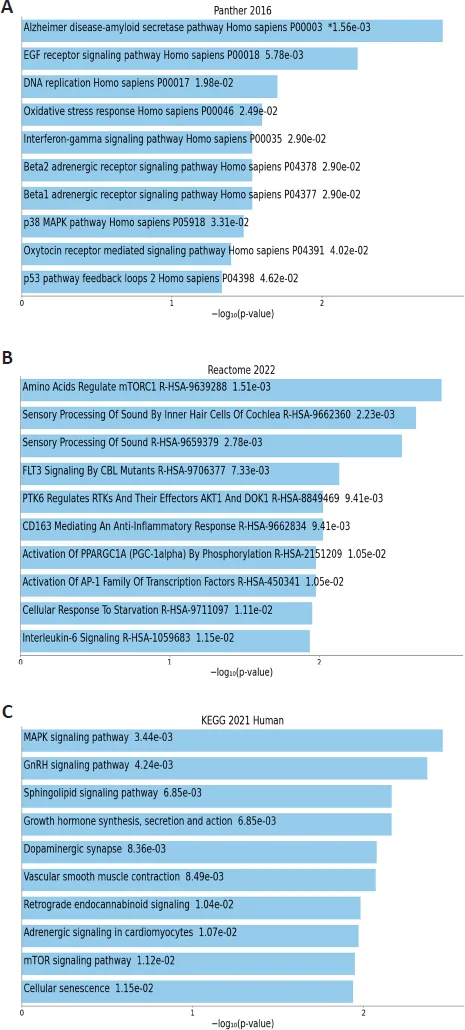
Terms associated with immune response, neuroinflammation, MAPK and mTOR signaling result highly represented after pathway enrichment analysis of single nuclei RNA hippocampal microglia in mice treated with NSC-sEV and MN-sEV. (A) Pathway enrichment analysis obtained from the PantherDB gene library. The analysis was made through EnrichR, a web-based gene-set enrichment analysis. The major terms identified are pathways related to AD (secretase pathway) and to immune response/neuroinflammation, such as Interferon-gamma signaling pathway, oxidative stress and p38 MAPK pathway, thus showing consistency with the bulk-RNA sequencing. (B) Pathway enrichment analysis obtained from the Reactome gene library, made with EnrichR. Here we see more pathways related to neuroinflammation/immune response, such as FLT3 signaling by CBL mutants, CD163 mediating an anti-inflammatory response, activation of PGC-1alpha, activation of AP-1 family of transcription factors, interleukin-6 signaling. These results appear converging towards the hypothesis whereby NSC-sEV induce microglia activation. (C) Pathway enrichment analysis obtained from the KEGG gene library, made with EnrichR. Here we can appreciate pathways related to MAPK signaling and mTOR signaling consistent with the involvement of *Fnip1*, *Fnip2*, and *Mapk14* as DEGs. (–log_10_ *P* represents the negative logarithmic function of *P*-value relative to the enrichment). mTOR: Mechanistic target of rapamycin.

Pathway analysis using PantherDB and Reactome (**Figures 6A** and **B**) identified the Interferon-gamma, Toll receptor signaling, JAK/STAT, p38MAPK, and TGF-β pathways, which align with the neuroinflammatory ones identified through bulk RNA sequencing. Notably, the AD amyloid secretase pathway was also enriched, suggesting that NSC-sEV may influence pathways directly associated with AD. Furthermore, the KEGG database (**Figure 6C**) identified the MAPK signaling pathway and mTOR signaling pathway as enriched.

## Discussion

This study investigated the effects of NSC-sEV on hippocampal microglia, revealing distinctive contributions of NSC-sEV to microglial function. While previous studies have shown that NSC-sEV from SVZ induce morphologic changes of microglia and regulate immune response and neuroinflammation (Morton et al., 2018), we demonstrate that although both NSC-sEV and MN-sEV are taken up by hippocampal microglia, only NSC-sEV treatment selectively enhances microglial activation, as evidenced by increased CD68 immunostaining. Importantly, NSC-sEV also lead to increased microglial uptake of TauO, indicating that these vesicles prime microglia for more efficient removal of neurotoxic molecules. This selective priming of microglia represents a novel mechanism through which NSC-sEV may promote immune surveillance and neuroprotection, distinguishing their effects from MN-derived sEV.

Specifically, we evaluated sEV internalization in hippocampal microglia, neurons, and astrocytes by injecting fluorescently labeled-sEV into the brains of wild-type mice and assessing their co-localization with each cell marker 24 hours post-administration. While no significant differences were observed between NSC-sEV and MN-sEV uptake within each cell type, microglia and neurons internalized most sEV in CA3 and CA1, compared with astrocytes; however, in the DG, neuronal uptake was significantly lower, and microglia remained the major recipient. Microglia have a higher internalization rate of extracellular vesicles (Pantazopoulou et al., 2023) and respond earlier to stimuli, compared with astrocytes, influencing microglia-astrocytes interactions that impact neuronal and synaptic functions (Jha et al., 2019). Our findings confirm that microglia play a major role in extracellular vesicle uptake, thus suggesting a role in the neuroprotective effect of NSC-sEV.

We next focused on microglia activation state upon sEV internalization by performing double immunostaining for Iba1, marker of total microglia, and CD68, marker of microglia activation. We observed increased levels of Iba1 in NSC-sEV recipient mice with significant differences in CA3 and DG between NSC-sEV and MN-sEV groups, indicating that more microglial cells are recruited after NSC-sEV injection. CD68 levels were significantly higher in NSC-sEV *vs* both naïve and MN-sEV in CA3, whereas in CA1 and DG, NSC-sEV mice displayed significantly higher CD68 levels with respect to naïve and PBS-treated animals, and a non-statistically significant difference between NSC-sEV and MN-sEV groups. These findings suggest that despite similar internalization rates, NSC-sEV induce higher microglial activation compared with MN-sEV, potentially leading to “primed” microglia, that protect neurons and synapses. Microglia are constantly surveilling the brain and removing non-functional synapses to maintain neuroplasticity, thus regulating neuronal activity and synaptic transmission (Schwabenland et al., 2021; Woodburn et al., 2021; Cornell et al., 2022). Although we found no difference in the uptake of NSC-sEV and MN-sEV by microglia, NSC-sEV appear to enhance the microglial response, which may contribute to the protective effects on neurons and synapses. Therefore, we propose that NSC-sEV enhance microglial immune surveillance activity in the hippocampus, thereby promoting microglial activation.

To further support these observations, we treated wild-type mice with TauO 24 hours post-sEV delivery and performed double immunostaining for Iba1 and TauO in the hippocampus. Previous studies show that Tau and TauO enhance microglia activation and phagocytosis (Das et al., 2020; Pereira et al., 2022; Taddei et al., 2023), perhaps through a mechanism involving EVs (Siew et al., 2024). Consistently, we observed that the microglia in NSC-sEV-treated mice showed increased uptake of TauO compared with MN-sEV-treated mice, thus indicating that NSC-sEV-induced microglial recruitment and activation may promote more efficient removal of toxic molecules from the CNS. Compelling evidence shows that an ICV injection of NSC-sEV prior to TauO delivery decreases accumulation of Tau in cortex and hippocampus (Krishnan et al., 2025). Similarly, *in vitro* studies on hippocampal neurons show that prior NSC-sEV treatment prevents TauO (Krishnan et al., 2025) internalization. Lysosomal dysfunction in neurons plays a crucial role in AD pathogenesis, leading to impaired autophagy and contributing to protein accumulation (Mançano et al., 2024; Chou et al., 2025). Recent evidence highlights that a variant autophagic receptor counteracts Tau accumulation in neurons, suggesting a potential protective mechanism against AD (Mattioni et al., 2025). Notably, NDAN individuals exhibit preserved autophagy and reduced hippocampal Tau levels, supporting autophagy crucial role in conferring resilience against cognitive decline (Tumurbaatar et al., 2023). Taken together with additional evidence showing a higher number of NSC (Briley et al., 2016) and more efficient microglia (Fracassi et al., 2023) in NDANs compared with AD individuals, our findings suggest that NSC-sEV modulate immune function and neuroinflammatory responses in microglia, mediating protective effects in the hippocampus.

Subsequently, we investigated the DEGs in hippocampal total homogenate and synaptosomes. In synaptosomes, NSC-sEV administration upregulates genes involved in synapse pruning, extracellular matrix, and regulation of immune effector process (NSC > PBS), which are linked to microglia (Gao et al., 2023), supporting our hypothesis. Conversely, MN-sEV administration upregulated pathways associated with cell activation and mitotic cell cycle (versus PBS), while genes regulating meiotic cell cycle, ion transmembrane transport, and regulating mast cell activation were upregulated in NSC-sEV compared with MN-sEV, without triggering immune response pathways. Noteworthy, the ontology analysis of the synaptosome preparation displayed enrichment of terms involving immune responses, such as “regulation of neuroinflammatory response” and “positive regulation of macroautophagy”. In the total homogenate preparation, NSC-sEV versus PBS showed upregulation of RNA splicing and DNA transcription pathways, while NSC-sEV versus MN-sEV highlighted genes involved in positive regulation of interleukin-6 production, and cytokine-mediated signaling pathways, thus confirming that NSC-sEV regulated the genes involved in the immune and neuroinflammatory response. Although microglia can be activated by “on” signals released by neurons, including MN-sEV (Bahrini et al., 2015; Pascual et al., 2020; Xia et al., 2022), in our investigations, MN-sEV modulated genes involved in metabolic or cellular pathways. Therefore, while NSC-sEV prime microglia towards a more reactive state, MN-sEV maintain microglia in a resting state, albeit in constant surveillance of the CNS (Eggen et al., 2013; Peng et al., 2021).

Next, we explored the direct effects of sEV on hippocampal microglia by snRNA-seq. Our analysis identified 21 different clusters in the hippocampus, each annotated to a specific cell type. We focused on the microglia cluster to identify DEGs and enriched pathways upon sEV administration. We observed upregulation of *Fnip1* and *Fnip2* (encoding FNIP1 and FNIP 2, folliculin interacting protein 1 and 2), and *Cbl* (encoding E3 ubiquitin-protein ligase CBL); conversely, we observed downregulation of *Slc7a8*, and *Mapk14*, which encode to LAT2, large amino-acid transporter 2 (also known as L-type amino acid transporter 2), and p38α MAP kinase 14, respectively. FNIP1 and FNIP2 form a complex with folliculin (FLCN) that regulates catabolism and anabolism at the lysosomes, through the modulation of mTORC1 and AMP-activated protein kinase (AMPK) (Ramirez Reyes et al., 2021). FLCN-FNIP1/2 is a positive regulator of mTORC1 and a negative regulator of AMPK, thus maintaining a balance between the two pathways to ensure cellular homeostasis. Both pathways are implicated in lysosomal biogenesis by modulating the transcription factors TFEB and TFE3; these factors bind to specific DNA sequences, regulating the expression of genes involved in lysosomal biogenesis, autophagy, immune, and stress responses (Ramirez Reyes et al., 2021). The mTORC1 signaling is amino acids-dependent and occurs on the lysosomal membrane, leading to reduced lysosomal biogenesis and autophagy (Valenstein et al., 2025). LAT2/SLC7A8, normally highly expressed in microglia (Braun et al., 2011), regulates the amino acid-dependent mTOR activation (Wang et al., 2022). However, in amino acid starvation conditions, the complex FLCN-FNIP is recruited to the lysosome membrane, thus inhibiting mTORC1 binding and promoting the formation of the lysosomal FLCN complex (Calvo et al., 2021; Ramirez Reyes et al., 2021). The TFEB/TFE3 then translocates to the nucleus and induces transcription of genes regulating lysosomal biogenesis and autophagy. Notably, the term ‘L-amino acid transmembrane transporter activity’ resulted downregulated in the bulk-RNA analysis, further supporting the findings from the snRNA seq.

Therefore, we hypothesize that the concomitant upregulation of *Fnip1* and *Fnip2* and downregulation of *Slc7a8* in hippocampal microglia upon NSC-sEV administration is suggestive of increased lysosomal biogenesis and overall higher phagocytic efficiency in the hippocampal microglia, perhaps through regulation of mTOR signaling. Further studies are needed to test this hypothesis.

Another gene regulating microglia activation and responsible for fine-tuning the immune response (Jafari et al., 2021; Augustin et al., 2023) when activated is *Cbl*; its inhibition leads to overproduction of proinflammatory cytokines with consequent excessive microglial activation, supported by NF-κB activation (Deng et al., 2025). Our results show upregulation of *Cbl*, suggesting that NSC-sEV -induced microglia activation is finely regulated to avoid excessive activation. Consistently, the downregulation of *Mapk14* gene suggests such fine-tuning of microglia activation; p38α is abundantly expressed in microglia (as well as the other p38MAPKs) and is responsible for microglia activation triggered by Aβ (Lin et al., 2022), thus promoting further production of pro-inflammatory cytokines and development of chronic inflammation (Chen et al., 2021). Moreover, p38α mediates Aβ-induced synaptic toxicity, further enhancing an unregulated neuroinflammatory response, which characterizes the progression of the neuropathology (Bachstetter et al., 2011; Falcicchia et al., 2020); conversely, microglial deletion or inhibition of p38α reduces trauma-induced pro-inflammatory damage in the brain, thus suggesting that its inhibition may lead to neuroprotection (Morganti et al., 2019; Luo et al., 2022; Liu et al., 2023; Son et al., 2023).

## Limitations

The study has some limitations that should be noted: the sEV fraction analyzed in this study was previously referred to as “exosomes” in our earlier work (Micci et al., 2019; Krishnan et al., 2025). In accordance with the latest guidelines from the International Society of Extracellular Vesicles (Welsh et al., 2024) we now refer to them as “small extracellular vesicles”. These latest guidelines also suggest investigating the presence of “EV corona” markers (Buzas, 2022) through WB. While we acknowledge the value of these new guidelines, we did not perform such analysis in the current study. Nevertheless, our characterization approach — including EM, nanoparticle tracking analysis, and WB for canonical EV markers and negative controls such as GM130 — is consistent with our previous publications (Micci et al., 2019; Krishnan et al., 2025), and with earlier field guidelines (Lötvall et al., 2014). We recognize this as a limitation and acknowledge the possibility of EV corona contributing to the observed biological effects.

## Conclusions

In conclusion, the increased microglial uptake of NSC-sEV which in turn leads to an enhanced TauO uptake following treatment, supports the hypothesis that NSC-sEV prime microglia for a more efficient immune response, likely contributing to the reduction of toxic protein aggregates associated with neurodegenerative processes. This aligns with previous observations showing that hippocampal NSC-sEV prevent synaptic Aβ oligomers binding (Micci et al., 2019) and that the brains of NDAN individuals, who maintain intact cognitive performance despite the presence of amyloid deposits (Bjorklund et al., 2012), have more NSC in the hippocampus DG compared with AD individuals (Briley et al., 2016), and contain more efficient microglia, capable of maintaining synaptic integrity (Fracassi et al., 2023). DEG analysis reveals that NSC-sEV regulate key pathways involved in lysosomal biogenesis, immune regulation, and neuroinflammation, suggesting their potential for the development of therapeutic strategies that modulate microglial activity and protect neuronal integrity in AD and related disorders. Future studies should focus on the mechanistic underpinnings of NSC-sEV -microglia interactions and their therapeutic potential for AD.

## Additional files:

***Additional Figure 1:*** *Quality control of small extracellular vesicles collected from NSC and MN cell culture.*

Additional Figure 1 Quality control of small extracellular vesicles collected from NSC and MN cell culture.
Characterization of small extracellular vesicles isolated from the conditioned medium of neural stem cells (NSC-sEV) and mature neurons (MN-sEV). (**A)** Representative high-resolution transmission electron microscopy image of small extracellular vesicles secreted from NSC and from MN (scale bars: 100 nm). (**B)** Nanoparticle tracking analysis of small extracellular vesicles isolated from NSC and MN shows a homogenous size distribution with a peak at 97 nm (NSC-sEV) and 103 nm (MN-sEV) (data is representative of 3 independent sEV preparations. (**C-E)** Total protein lysates of isolated small extracellular vesicles were analyzed by western blot for the presence of vesicular proteins, CD63, CD9, Hsp70, CD81, and the absence of the Golgi-associated protein GM130 as a control for cellular contamination, thus suggesting the enrichment of this fraction with sEV. Total mouse brain lysate was used as positive control. GM130: 130 kDa cis-Golgi matrix protein; Hsp70: heat shock 70 kDa; MN-sEV: mature neuron-derived small extracellular vesicles; NSC-sEV: neuronal stem cell-derived small extracellular vesicles.

***Additional Figure 2:*** *Western blotting of TauO preparation.*

Additional Figure 2 Western blotting of TauO preparation.
The image shows a scan of detected Tau oligomers after western blotting. Lane 2 and Lane 3: 15 μg of total protein from human AD brain homogenate; Lane 5: 0.5 μg of Tau oligomers; Lane 6: 1 μg of Tau oligomers. AD: Alzheimer’s disease; TauO: Tau oligomers.

***Additional Figure 3:*** *Different graphs obtained in Cellenics after alignment of single nuclei RNA sequencing with genome databases.*

Additional Figure 3 Different graphs obtained in Cellenics after alignment of single nuclei RNA sequencing with genome databases.
(A) After single-nuclei extraction and single-nuclei RNA sequencing, the data are aligned with genome databases, and further processed through several steps. Finally, a dimensionality reduction process results in UMAP graphs: in this example, 21 clusters were identified (with the platform Cellenics) by using the Louvain algorithm after principal component analysis. (B) After dimensionality reduction, the cluster can undergo a ScType annotation, which identifies the different clusters with specific cell types. Cluster 9 identified by the Louvain algorithm (see section A)) was annotated as “Microglial cells” by ScType method and was the focus of our analyses. (C) The same clusters described in A and B can be distributed based on the specific treatment that was administered to the mice. Although neuronal stem cell-derived small extracellular vesicles related clusters do not greatly differ from mature neuron-derived small extracellular vesicles related ones, it is still possible to tease out discrepancies in single cell populations. UMAP: Uniform Manifold Approximation and Projection.

## Data Availability

*All relevant data are within the paper and its Additional files.*
